# Reset Osmostat: A Challenging Case of Hyponatremia

**DOI:** 10.1155/2018/5670671

**Published:** 2018-11-04

**Authors:** Navin Kuthiah, Chaozer Er

**Affiliations:** Woodlands Health Campus 2, Yishun Avenue 2, Singapore 768024

## Abstract

Hyponatremia is the most common electrolyte abnormality seen in hospitalised patients with up to 15–20% of patients having a sodium level of less than 135 mmol/L (Reddy and Mooradian, 2009). Cases of hyponatremia were first described in the 1950s (George et al., 1955). As the differential diagnosis for hyponatremia is broad, a systematic and logical approach is needed to identify the cause. We describe a case of a 30-year-old gentleman who was found to have chronic hyponatremia. After a thorough workup, he was diagnosed to have reset osmostat. Reset osmostat is an uncommon and under recognised cause of hyponatremia which does not require any treatment. This diagnosis needs to be considered when the hyponatremia workup suggests SIADH, but the hyponatremia is not amenable to fluid restriction, salt or urea supplementation, and diuretic treatment.

## 1. Case Presentation

A 30-year-old gentleman with mild autism was admitted to hospital for a left supracondylar fracture following a fall. He was able to communicate and perform simple daily activities independently. He had a past medical history of epilepsy and allergic rhinitis. He was recently discharged from hospital about a week ago after being treated for pneumonia. His mobility was limited by poor vision. He had had multiple falls, some of them with head injuries. His regular medications included chloral hydrate, ferrous fumarate, calcium, vitamin D supplements, and sodium valproate. He did not smoke or drink alcohol [1, 2].

The fracture was treated conservatively. During the admission, his sodium was noted to be 128 mmol/L. Other blood test results are shown in Table 1. He was referred to the medical team for review when the sodium levels subsequently dropped to 120 mmol/L on day 3 of admission. Tracing his previous blood test results, his sodium has always been within the range of 124 to 126 mmol/L, and the chronic hyponatremia was previously attributed to psychogenic polydipsia. The previous tests done to investigate hyponatremia were not available for review. Physical examination did not reveal any significant findings. The patient was apyrexial, had a stable blood pressure of 125/80 mmHg with a heart rate of 80 beats per minute. He was clinically euvolemic. Postural blood pressure and heart rate measurements did not show any significant variation.

### 1.1. Investigations

As there was a drop in sodium levels from his usual baseline, the hyponatremia workup was repeated. His serum osmolality was 248 mOsm/kg, urine osmolality 387 mOsm/kg, and urine sodium 86 mmol/L. Thyroid function tests and 9 am cortisol levels were normal (Table 1). Urine osmolality of above 100 mOsm/kg suggested a degree of vasopressin secretion leading to inability to excrete free water.

The initial impression was SIADH secondary to sodium valproate, recently treated pneumonia, and pain from the left supracondylar fracture. A CT scan of the brain, thorax, abdomen, and pelvis performed to identify other causes of the hyponatremia was normal. As the patient had a urine osmolality of less than 500 mOsm/kg, he was initially placed on fluid restriction of 800 ml/day which was approximately 500 ml below his daily urine volume [3], but the serum sodium level remained between 120–125 mmol/L. He was then given 2 sodium chloride tablets 3 times per day. Each sodium tablet contained 600 mg of sodium chloride. His fluid intake was further restricted to 600 ml/day.

Despite these interventions, the sodium levels did not improve. He was also trialed on furosemide 20 mg twice daily. His sodium did increase to 130 mmol/L, but the patient was complaining of significant thirst, and his renal function deteriorated. He was subsequently taken off furosemide, and his sodium levels returned to his baseline of 126 mmol/L.

Due to the history of recurrent falls with head injuries, there was a possibility of cerebral salt wasting. However, the patient was clinically euvolemic and did not display any signs of dehydration at presentation. Also, the patient did not respond to sodium supplementation in the diet which goes against the diagnosis of cerebral salt wasting.

**Figure 1:**
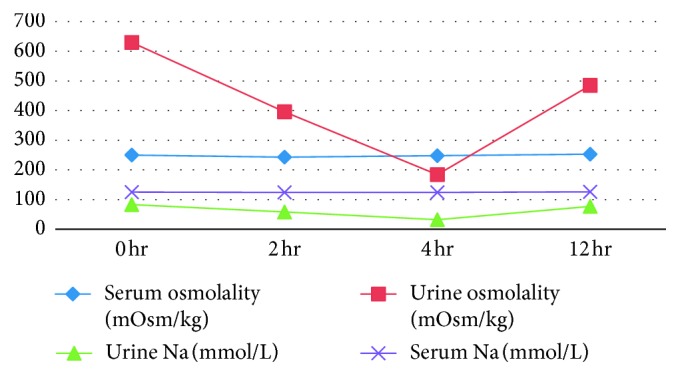
Water load test.

The possibility of reset osmostat was considered. A water load test was performed one week after cessation of diuretics. Following an overnight fast, the patient was given 800 ml of water (approximately 15 ml/kg) intravenously. About 720 ml of urine was excreted in 4 hours (220 ml at 1 hour, 340 ml at 2 hours, and 570 ml at 3 hours). The results are shown in Table 2 and Figure 1.

### 1.2. Treatment

A diagnosis of reset osmostat was made, and the patient was discharged without any sodium tablets and fluid restriction.

### 1.3. Outcome and Follow-Up

The patient remained clinically well and the sodium levels stable between 125–130 mmol/L. He is being followed up for 6 months in the clinic to monitor his sodium levels.

## 2. Discussion

It is essential to understand the pathophysiology of hyponatremia to appreciate the workup and management of the various causes of hyponatremia. Hyponatremia implies relative excess of free water compared to total body sodium. This can be caused by defect in renal water excretory mechanism or loss of sodium. The 2 main mechanisms which regulate water balance are thirst and vasopressin effect. Vasopressin which is released from the posterior pituitary is triggered by osmoreceptors and baroreceptors. Vasopressin acts on the aquaporin 2 water channels of the apical membrane of the collecting duct increasing water permeability leading to increased water reabsorption.

Hyponatremia can be classified in many ways. The objective of classification is to enable easy workup and approach to hyponatremia (Table 3).

Volume status assessment should be performed routinely on all patients with hyponatremia. However, the assessment of volume status is often challenging especially in patients with obesity and those with preexisting heart or renal failure [4, 5] (Table 4).

Acute hyponatremia needs to be addressed promptly, whereas chronic hyponatremia can be investigated before the appropriate management is instituted. The symptoms of hyponatremia can vary from mild symptoms such as balance disturbance, headache, and lethargy to severe symptoms such as seizures, confusion, vomiting, and drowsiness due to cerebral oedema. Severe symptoms do not manifest until the sodium level is below 120 mmol/L [6]. Distinguishing between acute and chronic hyponatremia cannot always be done based on symptomatology.

Patients with chronic hyponatremia may appear minimally symptomatic or asymptomatic. However, recent studies have suggested hyponatremia is always symptomatic, but the symptoms may be subtle and may not be picked up on routine history taking and physical examination [7]. In vitro studies have implicated increased osteoclast activity, and analysis has proved that hyponatremia is a clinically important risk factor for osteoporosis and fracture [8]. Hyponatremia is also associated with increased risk of falls [9].

Fluid restriction is useful in SIADH, heart failure, chronic kidney disease, liver disease, and primary polydipsia as the aim of treatment is to remove excess free water from the body. The daily water intake must be lowered beyond daily water losses [10]. A loop diuretic may be effective in reducing the relative water excess if fluid restriction alone is ineffective [11]. Hypertonic saline may be administered for SIADH. However, due to the risks of rapid overcorrection of the hyponatremia and pulmonary oedema, it is reserved for patients who are symptomatic [12]. Oral salt tablets can be also given, but it may be difficult to consume the recommended 200 mmol of sodium chloride daily. The main aim of sodium administration either intravenously or via the oral route is to induce osmotic diuresis. Free water is excreted along with sodium via the kidneys as the renal handling of sodium is normal. Demeclocyline and lithium improve sodium levels by blocking the action of vasopressin on the kidney tubules. These drugs are not frequently used because of unfavourable side effect profiles. The response to these drugs is also slow and variable [13]. Renal vasopressin receptor inhibitors have shown promise in the management of SIADH. The medications are now available in tablet form and have an acceptable side effect profile [14].

Reset osmostat is under recognised and is often not reported. Some authors claim that 36% of patients with SIADH have reset osmostat [15]. Reset osmostat is classified as type C SIADH [16]. The etiology of reset osmostat is unknown. However, associations with tuberculosis, alcoholism, pregnancy, psychogenic polydipsia, psychosis, and gastric, colonic, and oat cell carcinomas and the elderly have been reported [17]. The course of illness is variable with reset osmostat persisting for long periods of time, but some cases resolve spontaneously or after treatment of the underlying condition [18]. Vasopressin is released once the osmoreceptors in the hypothalamus detect an increase in serum osmolality or the baroreceptors detect hypovolaemia. The normal osmostat for vasopressin release is fixed between 275 and 295 mOsm/kg. When the serum osmolality is below 280 mOsm/kg in normal individuals, vasopressin levels are very low [19]. In reset osmostat, this set point for vasopressin release is decreased meaning vasopressin is released at a lower than normal serum osmolality threshold [19]. This causes increased water conservation by the kidneys leading to lower serum osmolality and hyponatremia. The hyponatremia in reset osmostat is often mild, and the patients are frequently asymptomatic. The hyponatremia does not progress below the osmolality at which the osmostat has been reset. Attempting to correct the sodium levels will increase plasma osmolality which will in turn trigger the secretion of vasopressin [7]. It is important to diagnose reset osmostat in order to avoid further unnecessary investigation and treatment. Attempting to raise the sodium levels above the new reduced baseline will lead to increased vasopressin levels and thirst [19]. There are no studies or case reports to indicate if patients with reset osmostat display any subtle manifestations which may be attributed to the persistent hyponatremia. If patients with reset osmostat require intervention, it would prove challenging to treat. Based on the understanding of the pathophysiology of the disease, agents which block the effect of vasopressin in the collecting ducts may hold the key to correcting the hyponatremia. This however has not been studied. Reset osmostat should be considered in patients with chronic mild to moderate hyponatremia. The hyponatremia workup in reset osmostat is identical to SIADH where serum osmolality is low with elevated urine osmolality and sodium levels. The water load test can be used to differentiate reset osmostat from SIADH.

It should only be performed after other causes of SIADH are fully worked up and the diagnosis of reset osmostat is strongly suspected. The test should be performed under close supervision as it carries a risk of worsening hyponatremia and fluid overload. The patient is given a water load of 10 to 15 ml/kg orally or intravenously. Normal individuals and those with reset osmostat should excrete more than 80% of the water load within 4 hours [20]. The volume of fluid administered for the water load test varies between studies. In a study of 4 patients with chronic and stable hyponatremia, 20 ml/kg of water load was administered. In each case, more than 80% of the water load was excreted in 4 hours for the diagnosis of reset osmostat to be made [19]. There are no studies to validate the water load test.

The rationale behind the water load test is to determine the ability of the body to appropriately suppress vasopressin levels in response to a water load which reduces serum osmolality. In SIADH, the excess vasopressin levels would not be suppressed despite a reduction in serum osmolality leading to more fluid retention and a further reduction of serum osmolality and serum sodium. Thus, the water load test must be performed with caution as it may aggravate the hyponatremia in patients with other subtypes of SIADH. Checking vasopressin levels would not have aided in differentiating between SIADH and reset osmostat. Furthermore, the vasopressin levels are influenced by numerous factors such as medications, posture, diurnal variation, and discrepancies between normal lab values makes the interpretation challenging.

A small study has explored the role of fractional excretion of urea in the diagnosis of reset osmostat. The diagnosis of reset osmostat was made in nonoedematous hyponatremic patients with normal fractional excretion of urate levels, suggesting that a normal fractional excretion of urea is a good indicator of reset osmostat regardless of urine sodium or serum urate levels [7]. Patients with other subtypes of SIADH have increased fractional excretion of urate levels. However, it is unclear whether this method can replace the traditional water load test in arriving at the diagnosis of reset osmostat.

## 3. Conclusion

Although hyponatremia is very commonly seen in clinical practice, it often presents a diagnostic challenge to doctors. A systematic approach is needed to identify the underlying cause. Whilst most causes of hyponatremia need urgent attention and correction, reset osmostat does not need any intervention. The knowledge available on reset osmostat is limited as there are very few studies published. More research needs to be performed to understand the course of the disease and possible subtle manifestations of hyponatremia.

## Figures and Tables

**Table 1:** Blood test results.

Hb	13.2 g/dL (13–18)	TSH	3.2 mU/L (0.35–5.50)
WBC	6.4 × 10^9^/L (4–11)	T4	13 pmol/L (10.5–20)
Plt	457 × 10^9^/L (150–500)	9 am cortisol	487 nmol/L (180–620)
Na	128 mmol/L (134–145)	LFTs	Normal
K	4.2 mmol/L (3.5–5)	Ca (total)	2.5 mmol/L (2.12–2.65)
Ur	6.7 mmol/L (2.5–6.7)	Phosphate	0.9 mmol/L (0.8–1.45)
Cr	88 mmol/L (70–150)		

**Table 2:** Result of the water load test.

Time	0 hr	2 hr	4 hr	12 hr
Serum osmolality (mOsm/kg)	250	243	248	253
Urine osmolality (mOsm/kg)	630	396	184	485
Urine Na (mmol/L)	83	58	32	77
Serum Na (mmol/L)	125	124	124	126

**Table 3:** Classification of hyponatremia.

(1) Biochemical severity
(a) Mild hyponatremia, 130–135 mmol/L
(b) Moderate hyponatremia, 125–129 mmol/L
(c) Profound hyponatremia, <125 mmol/L
(2) Time of onset
(a) Acute hyponatremia <48 hours
(b) Chronic hyponatremia >48 hours
(3) Symptoms
(a) Symptomatic hyponatremia
(b) Asymptomatic hyponatremia
(4) Volume status
(a) Hypovolaemia
(b) Normovolaemia
(c) Hypervolaemia
(5) Serum osmolality
(a) Hypotonic hyponatremia, <275 mOsm/kg
(b) Isotonic hyponatremia, 275–295 mOsm/kg
(c) Hypertonic hyponatremia, >295 mOsm/kg

**Table 4:** Assessment of volume status.

Increased volume	Decreased volume
Gallop rhythm (11)	Increase capillary refill time (6.9)
Elevated JVP (5.1)	Sunken eyes (3.4)
Leg oedema (2.3)	Dry axilla (2.8)
Hypotension (2.0)	Dry tongue/mucus membranes (2.1)
Wheeze (0.5)	Postural pulse increase (1.7)
Ascites (0.33)	Postural hypotension (1.5)

Values in brackets are positive likelihood ratios.
